# Greater Subjective Well-Being Associated With Lower Inflammatory Proteins in an Older Adult Sample From the NHATS

**DOI:** 10.1093/geroni/igaa057.1977

**Published:** 2020-12-16

**Authors:** Melissa Hladek, Shang-En Chung, Thomas K M Cudjoe, Laura Samuel, Sarah Szanton, David Roth

**Affiliations:** 1 Johns Hopkins University, Baltimore, Maryland, United States; 2 Johns Hopkins University School of Medicine, Baltimore, Maryland, United States

## Abstract

Subjective well-being (SWB), comprised of cognitive and affective evaluations of life, is associated with better health outcomes and lower mortality, but mechanisms are poorly understood. We examine the associations between SWB and its subscales with two biomarkers: Interleukin-6 (IL-6) and C-Reactive Protein (CRP), both common inflammatory indicators associated with mortality and increased cardiovascular disease. Dried blood spot data collected from 4,648 older adults NHATS participants in 2017 was used. After adjustment for age, sex, race/ethnicity, education, tobacco, body mass index and chronic disease, we found greater SWB and greater scores on subscales including positive affect, self-realization and personal mastery were all significantly associated with decreased IL-6 and CRP. Conversely, increases in negative affect was significantly associated with increased IL-6 and CRP values. This study adds evidence of a potential mechanistic mind-body connection pathway.

